# Supplementary material for: Parents, Teachers, and Community: A Team Approach to Developing Physical Competence in Children
Source: Children (Basel). 2023 Aug 9;10(8):1364. doi: 10.3390/children10081364 (PMC10453419; doi:10.3390/children10081364)
Supplement: Supplementary file 1 [file children-10-01364-s001.zip › children-2487567-supplementary.pdf]

Supplementary Material Figure S1 – Instructional Card

Community Initiatives Fund

University of Saskatchewan
College of Kinesiology

Wacky Walks

How to Play:

1. Choose a destination like the corner store, the park or the community centre.
2. Travel in as many different ways as possible between light posts, fence posts, or other markers on the way to your destination. Choose a new form of movement at each marker!
3. Use movements like gallop, skip, run, side-shuffle, or crossovers. Use the curb to balance like a tightrope walker! Bring the skipping rope and see if you can skip while moving forward!
4. When you run out of ways to move, challenge yourselves to do these movements in slow-motion or high-speed.

Equipment:

- Skipping Rope (optional)

Developing Skills:

- Gallop
- Skip
- Jump
- Run
- Balance

Cues:

- Remember that galloping is "heel-toe, heel-toe"
- Remember that skipping is "step-hop, step-hop"
- Lift your knees and swing your arms when skipping
- Make a T shape with your arms for better balance

Building Confidence, Competence and Motivation

1. Which part of the walk was your favourite? Why?
2. Which part of the walk was your least favourite? Why?
3. Do you ever move like this at school or when you are playing with your friends? Do you think that you could?
4. What is the difference between a skip and a gallop?

Check out the following website for video examples of these skills:
http://fms.60minkidsclub.org/?page_id=270

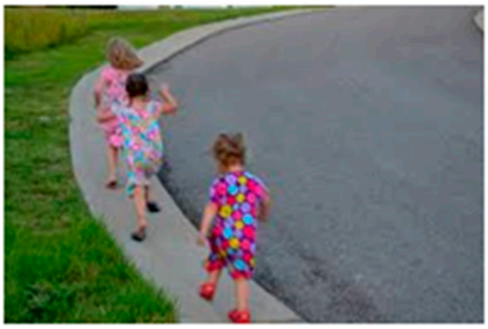

© University of Saskatchewan Physical Literacy Team (USPLIT)

Supplementary Material Figure S2 – Lesson Plan

Spatial Awareness

Curriculum Outcomes

PE1.6
Apply an understanding of how to vary the movement of the body while performing locomotor, non-locomotor, and manipulative skills through changes in:

- space (personal space, general space, levels, directions, pathways)

PE1.9
Demonstrate, with little or no support, safe and cooperative behaviours while participating in physical education activities.

PE1.10
Communicate and demonstrate an understanding of self-control, a consideration for others, and a respect for differences among people (e.g., abilities, interests, likes and dislikes, gender, culture) while participating in physical education settings.

Equipment:

- bean bags (enough for 1 per student)
- 10 hula hoops
- tambourine or music – something to stop and start motion

Warm Up: (4 min.)

Equipment: none

Space Invaders (Students need to spread out and fill the space.)**:** Before beginning the teacher will tell the students that they must stay inside the black line of the basketball court (Games are played with boundaries, so learning to play within boundaries is important. Also, it creates safe boundaries, as walls are not boundaries for most activities.) The students will begin by walking around in their own personal space. When the teacher signals the students to "stop" the students need to be in their own space (not near anyone or anything – walls, equipment). If students are successful, increase the pace of movement to a jog.

Note: The signal to "stop" could be stopping music that is playing or banging the tambourine.

Skill Development: (15 min.)

The teacher will explain what "personal and general space" is. *Personal space* - physical space immediately surrounding someone and *General space* - is the space within a room or bounded area that a person can move through using any means of locomotion.

Equipment: 1 bean bag per student

1. **Islands –** The students will take a beanbag and place it in its own spot inside the basketball court boundary in the gym. Students will place 1 foot on their bean bag. The teacher should instruct the students to pay attention to where their spot is (near a line, inside a circle on the floor, etc.). The teacher will instruct the students to move about the gymnasium in "general space" and away from their bean bag. When the teacher calls "Islands" the students are to return as quickly and as safely as

possible to their own bean bag. The teacher can change ways, levels and pathways to travel, based on their experiences.

Equipment: about 10 hula hoops

2. **Cooperative Hoops** – The teacher will scatter 10 hula hoops throughout the basketball court. The students will be instructed to move around the gym in general space, but in their own personal space, as they are able to, walk, run, etc. When the teacher indicates (stoppage of music, hit the tambourine) the students will go to the nearest hoop and put one foot in it. Based on where students are at the "stoppage time" they may be sharing a hoop with a number of other students or they may have a hoop to themselves. Repeat numerous times.
   Note: The teacher can take away hula hoops so that the students have to cooperate a little bit more. Never go below 2 and keep the hoops at the opposite ends of the gym.

Culminating Activity: (5 min.)

Equipment: none

DVD Player – The teacher will instruct the students to move around the gym in general space, but in their own personal space, as they are able to, walk, run, etc. when she/he says "PLAY". The teacher will give **a** command:

- Pause – they **freeze**
- Fast forward – they move around **forward** quickly
- Rewind – they move **backwards** (walking slowly)
- Slow Motion – they move around very **slowly**

After a few seconds the teacher will say "PLAY" and the students will resume moving forward safely. The teacher will then give another command. This will go on for 5 minutes.

Closure: (2 min.)
Review with the students what "personal" and "general" space is.

© University of Saskatchewan Physical Literacy Team (USPLIT)
